# Air pollution and respiratory diseases in conurbated cities in the state of São Paulo

**DOI:** 10.1590/1984-0462/2025/43/2024178

**Published:** 2025-06-27

**Authors:** Luiz Fernando Costa Nascimento, Ana Clara Silva Raposo de Almeida, Julia Ferreira Gomes Pereira, Mariana Telles de Castro, Adriana Oliveira Ribeiro dos Santos

**Affiliations:** a Universidade Estadual Paulista – Guaratinguetá (SP), Brazil.; b Universidade de Taubaté – Taubaté (SP), Brazil.

**Keywords:** Respiratory diseases, Air pollutants, Particulate matter, Child health, Doenças respiratórias, Poluentes atmosféricos, Material particulado, Saúde da criança

## Abstract

**Objective:**

To estimate the association between exposure to particulate matter with less than 10u of aerodynamic diameter (PM_10_) and hospital admissions due to acute respiratory diseases in children.

**Methods:**

This is an ecological time series study with data on hospitalizations for respiratory diseases in children living in the conurbated cities of Taubaté, Tremembé, and Pindamonhangaba located in the Vale do Paraíba in São Paulo. Hospital admission data refer to the period between January 1, 2016 and December 31, 2019. Information on hospital admissions was obtained from the DATASUS database, and concentration values (μg/m^3^) of the pollutant PM_10_, temperature, and humidity relative air were obtained from the Environmental Company of the State of São Paulo. Negative binomial regression was used. Population attributable fraction and hospitalization costs were estimated.

**Results:**

A total of 1,291 hospitalizations were identified in the conurbated municipalities with a daily average of 0.88 standard deviation (±) 1.06, varying between 0–7 hospitalizations. Significant exposures to PM_10_ can be observed at all lags, except the four-day lag (Lag 4). The total cost of hospitalizations was around US$ 800,000.00 and the excess number of hospitalizations (135) represented an expense of around US$ 80,000.00.

**Conclusions:**

Studies with conurbated cities are easily applicable, extending the study area. The results reinforce the harmful role of exposure to air pollutants in children’s health.

## RESUMO

**Objetivo:**

Estimar a associação entre exposição ao material particulado poluente com diâmetro aerodinâmico inferiores a 10u (PM_10_) e internações hospitalares por doenças respiratórias agudas em crianças.

**Métodos:**

Estudo ecológico de série temporal com dados de internações por doenças respiratórias em crianças residentes nas cidades conurbadas de Taubaté, Tremembé e Pindamonhangaba localizadas no Vale do Paraíba paulista. Os dados de internações hospitalares referem-se ao período entre 1º de janeiro de 2016 e 31 de dezembro de 2019. As informações das internações hospitalares foram obtidas no banco de dados DATASUS, e os valores de concentração (ug/m3) do poluente PM_10_, temperatura e umidade relativa do ar foram obtidas junto à Companhia Ambiental do Estado de São Paulo. Foi utilizada regressão binomial negativa. A fração atribuível populacional e os custos de internação foram estimados.

**Resultados:**

Foram identificadas 1.291 internações nos municípios conurbados com média diária de 0,88 desvio padrão (±) 1,06, variando entre 0–7 internações. Exposições significativas ao PM_10_ podem ser observadas em todas as defasagens, exceto na de quatro dias (Lag 4). O custo total das internações foi em torno de US$ 800.000,00 e o excesso de internações (135) representou um gasto em torno de US$ 80.000,00.

**Conclusões:**

Estudos com cidades conurbadas são facilmente aplicáveis aumentando a área de estudo. Os resultados reforçam o papel nocivo da exposição aos poluentes atmosféricos na saúde infantil.

## INTRODUCTION

Air pollution is a global topic and a health concern. The burden that air pollution places on national healthcare systems is also growing. According to the World Health Organization, around 4.2 million deaths per year are attributable to ambient air pollution. Cardiac diseases, strokes, pulmonary cancer, and chronic pulmonary diseases account for the vast majority of these deaths.^1^

Respiratory diseases in children were responsible for 1.75 million hospital admissions in the Unified Health System (SUS) in Brazil, generating costs of around R$1.4 billion (≈ US$450 million) accounting for just over 9,000 deaths. In the state of São Paulo, there were 360,000 hospitalizations with costs of just over R$330 million (≈ US$100 million) and responsible for 1,500 deaths between 2016 and 2019.^2,3^

An extensive review of literature concluded that exposure to air pollution is associated with an increased risk of respiratory hospital admissions in children worldwide. Particulate matter with aerodynamic diameter ≤10 μm (PM_10_) and ≤2.5μm (PM_2.5_), sulfur dioxide (SO_2_), carbon monoxide (CO), nitrogen dioxide (NO_2_), and ground-level ozone (O_3_) are associated with increased hospital admissions for many causes among children; the burning of fuel by the vehicle fleet is a major source of air pollution.^4,5,6,7^

Studies carried out in medium-sized Brazilian cities identified an association between exposure to air pollutants and hospitalizations for respiratory diseases.^8,9,10,11^ In these studies, temperature was included in the model as a control variable. Children and elderly people are the most affected age group due to respiratory diseases associated with exposure to air pollutants.

The objective of the present study is to estimate the association between exposure to PM_10_ and hospital admissions due to acute respiratory infection diseases in children under 10 years old residents in Taubaté, Tremembé, and Pindamonhangaba located in Vale do Paraíba, state of São Paulo, between 2016 and 2019.

## METHOD

**Figure 1:**
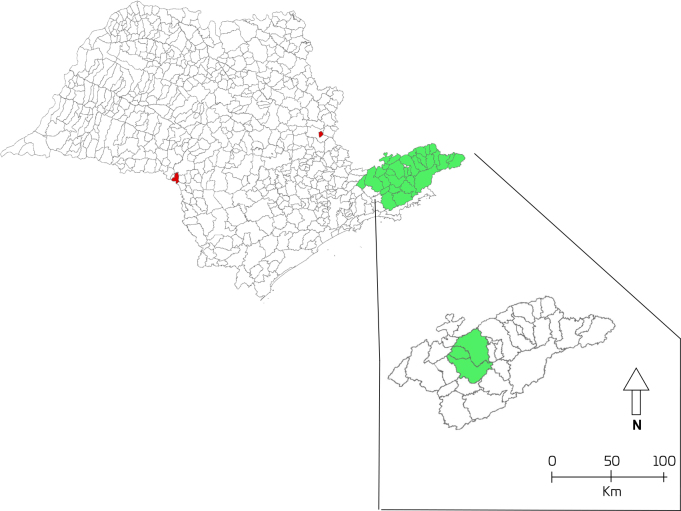
Map of the state of São Paulo with emphasis on Vale do Paraíba and the agglomerated municipalities.

This is an ecological time series study with data on hospitalizations for respiratory diseases in children living in the agglomerated cities of Taubaté, Tremembé, and Pindamonhangaba located in the Vale do Paraíba in São Paulo state. These conurbated municipalities have their coordinates 23° South and 45° West, with a population of approximately 530,000 inhabitants in an area of 1,500 km^2^ (Figure 1).

The available information on hospital admissions were obtained from the public, open-access Brazilian database DATASUS^8^ referring to diagnoses J04, J12–J18, J20–J21, and J45 of the International Classification of Diseases 10th revision (ICD10) for ages up to 10 years.

The concentration values (μg/m^3^) of the pollutants PM_10_, O_3_, and NO_2_, and also of meteorological variables such as maximum and minimum daily values of temperature (°C), and humidity relative air content (%) were obtained from the Environmental Company of the State of São Paulo (CETESB).^7^ The daily temperature amplitude was determined by the difference between the daily maximum and minimum values.

Hospital admission data refer to the period between January 1, 2016 and December 31, 2019. As they are counting data, the probability distribution that most resembles this distribution of values is the Poisson. If the average value of hospitalizations is close to the variance value, the linear Poisson regression model can be used; otherwise, the negative binomial approach is applied.

The regression models used the concentrations of the three pollutants simultaneously, as well as the values of maximum temperature, relative air humidity, and day of the week. In another step, the thermal amplitude was inserted. In another model, maximum temperatures were replaced by minimums, but the other variables were maintained to identify whether the relative risk of exposure to pollutants could be modified by different temperatures.

As the effects of exposure to air pollutants can manifest themselves not only on the same day of exposure, but on subsequent days, lags of zero (hospitalization on the same day of exposure) – Lag 0 and hospitalizations up to five days after exposure were constructed (Lag 5) and this window is not yet very well defined.

The link function is the logarithmic function ln (HA); where HA is the hospital admission expected value. The corresponding equation is as follows:


ln(HA) =β0+β1(POL)+ +β2(RH) +β3(TEMP) +β4(DOW) +β5(RT)

The relative risk (RR) was estimated as RR = exp(β*D(conc-pol)); where β is the coefficient provided by Poisson regression or negative binomial regression for a given pollutant, and Δ(conc-pol) is the increase in pollutant concentration, commonly 10μ/m^3^. RH is the relative air humidity, TEMP stands for temperature, DOW for day of week, and RT is range of temperature.

Then, it is possible to obtain the proportional attributable ratio (PAR) according to the expression PAR = 1 – (1/RR). With this PAR value, it is possible to calculate the population attributable fraction (PAF) according to the expression PAF = PAR*N; where N is the total outcome studied, in this case, hospitalizations for respiratory diseases in children.

The estimated coefficient values for PM_10_, according to the maximum or minimum temperatures included in the model, were compared according to the expression $\text{z}=\left(\beta 1-\beta 2\right)/\sqrt{{\left(E1\right)}^{2}+{\left(E2\right)}^{2}}$ ; where β1and β2 are the estimated values of the coefficients, and (E_i_)^2^ is the square of the average error value of each coefficient.

The costs of these hospitalizations for the SUS were estimated. This information is also available at the same address that provides information about hospitalizations.

As this is an ecological study using secondary data available to the public, submission to the Ethics Committee was waived.

## RESULTS

During the study period, 1,291 hospitalizations were identified in the agglomerated municipalities of Taubaté, Tremembé, and Pindamonhangaba with a daily average of 0.88 standard deviation (±) 1.06, varying between 0–7 hospitalizations. The mean values and their respective standard deviations and minimum and maximum values of the study variables are presented in Table 1. The missing values of the other variables did not compromise the analyses as they were not sequential.

**Table 1:** Meteorological variables in mean, standard deviation, minimum, and maximum values. Taubaté, Tremembé and Pindamonhangaba, 2016–2019.

	Mean (SD)	Minimum–Maximum
PM_10_ (μg/m^3^) (27)*	23.4 (11.8)	4.0–72.0
NO_2_ (μg/m^3^) (82)	35. 4 (16.5)	2.0–112.0
O_3_ (μg/m^3^) (8)	73.4 (23.2)	8.0–169.0
MaxT (°C) (12)	28.2 (4.3)	13.2–37.5
MinT (°C) (13)	15.5 (4.4)	1.7–22.8
RH (%) (7)	47.1 (14.2)	12.0–92.0
Range (°C) (8)	12.6 (4.8)	4.1–23.8

PM_10_: particulate matter with less than 10u of aerodynamic diameter; NO_2_: nitrogen dioxide; O_3_: ozone; MaxT: maximum temperature; MinT: minimum temperature; RH: relative air humidity; SD: standard deviation.

*missing data.

Hospitalizations were positively correlated with PM_10_ concentrations and negatively correlated with maximum and minimum temperatures (data not shown).

**Figure 2:**
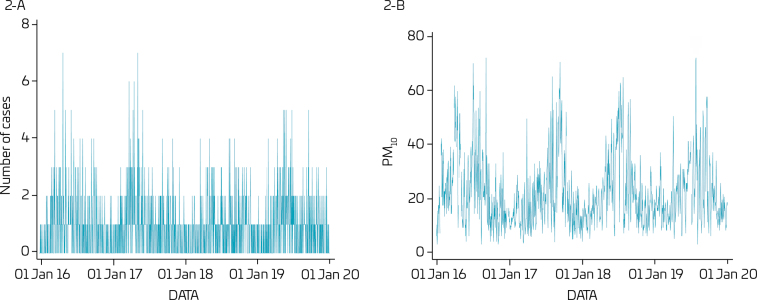
Daily values for the number of hospitalizations (cases) (2A) and concentrations of particulate matter (PM_10_) (2B). Taubaté, Tremembé, and Pindamonhangaba, 2016–2019.

Daily distribution of cases and PM_10_ concentrations are revealed in Figures 2A and 2B, that show a seasonal distribution with higher values in the months of May to August, which are colder and have a lower percentage of relative air humidity.

The values obtained from the negative binomial regression for the PM_10_ analyzed with the pollutants NO_2_ and O_3_ for Lags 0–5 are exhibited in Table 2, with the inclusion of maximum and minimum daily temperatures. Analyses involving daily temperature ranges did not show significant differences and are not described in tables.

**Table 2:** PM_10_ coefficient value provided by regression with standard deviation, according to lags from zero to five days with the presence of maximum and minimum temperatures in the model. Taubaté, Tremembé, and Pindamonhangaba, 2016–2019.

	Maximum temperature (SD)	Minimum temperature (SD)
LAG 0	**0.007998 (0.003450)**	**0.008087 (0.003466)**
LAG 1	**0.011079 (0.003391)**	**0.011009 (0.003421)**
LAG 2	**0.009547 (0.003421)**	**0.009334 (0.003458)**
LAG 3	**0.007701 (0.003481)**	**0.007531 (0.003518)**
LAG 4	0.004277 (0.003532)	0.004217 (0.003557)
LAG 5	**0.008128 (0.003470)**	**0.007824 (0.003497)**

PM10: particulate matter with less than 10u of aerodynamic diameter; SD: standard deviations (in parentheses). Values in bold: p<0.05.

Significant exposures to PM_10_ can be observed at all lags, except Lag 4, depending on the maximum or minimum daily temperatures. When comparing the estimated coefficients for exposure to PM_10_, there were no statistically significant differences between them, suggesting that temperature does not maximize or minimize the effects of exposure to PM_10_ on hospitalizations for the respiratory diseases selected in this study.

The maximum RR value of exposure to PM_10_ according to an increase of 10μg/m^3^ was RR=1.12. The estimated PAR value, according to the highest RR value, was 0.105 (10.5%). In this situation, the estimated PAF value was 135 hospitalizations. The total cost of hospitalizations, both in the ward and the intensive care unit, according to the selected diagnoses over the four years, with an average hospitalization cost of around R$2,300.00, was around R$3 million (≈ US$ 800,000.00). The excess number of hospitalizations (135) represented an expense of around R$312,000 (≈ US$ 80,000.00) for the SUS.

**Figure 3:**
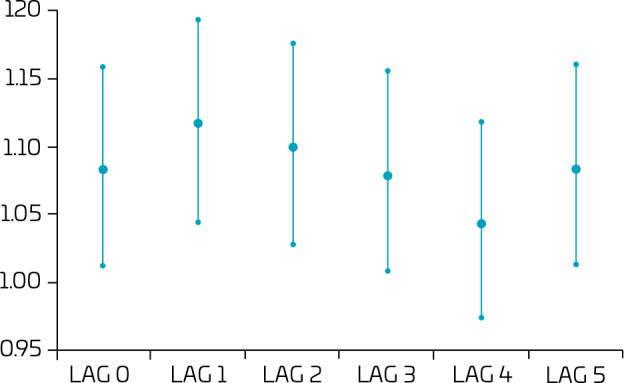
Relative risk of hospitalizations, according to increase of 10 μg/m^3^ PM_10_, and lags of 0 to 5 days. Taubaté, Tremembé, and Pindamonhangaba, 2016–2019.

Relative risk according to lag distribution is shown in Figure 3.

## DISCUSSION

The present study, using conurbation data from three municipalities located in Vale do Paraíba in São Paulo, found an association between exposure to PM_10_ and hospitalizations of children diagnosed with some respiratory system diseases.

There was no difference in the risk of hospitalization due to respiratory diseases in children associated with exposure to PM_10_ when analyzing the maximum and minimum temperatures in different models.

An association between exposure to PM_10_ and hospitalizations due to respiratory diseases in children was found, also in Vale do Paraíba, in a study carried out 20 years ago when PM_10_ concentrations were around 44 μg/m^3^, practically double of those found in the present study.^9^

Nguyen et al.^1^ found, in Hanoi (Vietnam), a PM_10_ average concentration of 80 μg/m^3^ and a 0.6% increase in hospitalizations for pneumonia in an age group from 0 to 15 years resulting from an increase of 10 μg/m^3^ in PM_10_ concentrations. This difference can be explained by the way in which data from Hanoi or variables from the children population were analyzed that were different from our children population. Several factors can contribute to these differences, including regional climate variations, land use patterns, and local policies such as transportation, industry, and urbanization.

Tran et al.^10^ also pointed out an association between exposure to particulate matter, both PM_10_ and the fine fraction, PM_2.5_, and pneumonia in children with risks similar to those found in our study. These authors draw attention to climate change and its extreme events that could substantially affect susceptible populations.^10^

A study carried out in Santiago (Chile), involving 72,000 children up to 15 years old diagnosed with respiratory diseases (J00–J99 according to ICD10), with data from March to September in the years between 2001 and 2005, noticed an increase between 8–11% proportionately to the increase in PM_2.5_ concentrations, the average of which was 41.2 μg/m^3^ (the average for PM_10_ was 81.5). An increase of 10 μg/m^3^ in the concentrations of this pollutant was responsible for the increase of 8–11% in hospitalizations for pneumonia, bronchiolitis, and asthma. PM_2.5_ was not included in our study due to significant missing data which could compromise the results.^11^

Seasonal influence, particularly in winter, has been investigated due to potential risks to children’s health, especially in relation to the respiratory system such as asthma, pneumonia, bronchitis/bronchiolitis, and influenza, among other diseases. During this period, children may be confined for longer periods, with greater circulation of some viruses, such as respiratory syncytial virus (RSV); in addition, low temperatures promote respiratory tract spasms and ischemia due to capillary contraction in children, resulting in weakened ciliary movement and, hence, weakened removal of pathogens, such as RSV in the respiratory epithelium.^12,13,14^

The mechanisms of action of these pollutants are still poorly understood and, among them, it may be highlighted the production of free radicals of oxygen and nitrogen resulting from high concentrations of particulate matter, ozone, and nitrogen oxides, which initiate an inflammatory response, with the release of mediators such as cytokines that lead to effects on the respiratory system.^15^

The connection between air pollution and respiratory diseases is complex, involving multiple physiological pathways that can lead to inflammation, oxidative stress, and changes in the immune system. Consequently, PM_2.5_ exposure can generate reactive oxygen species, which can provoke oxidative stress and damage to lung tissue, exacerbating the development of respiratory diseases by inducing oxidative stress, enhancing pro-inflammatory responses, and compromising the airway epithelial barrier function, thus sensitizing the lung to allergic antigens and hindering the lungs’ ability to remove pollutants.^16^

National studies^17,18^ usually use data from state environmental agencies; however, where these sources are not available, a viable alternative is to use data provided by mathematical models such as the Coupled Chemistry Aerosol-Tracer Transport model to the Brazilian developments on the Regional Atmospheric Modeling System (CCATT-BRAMS). CCATT-BRAMS is a mathematical model that performs numerical simulations of weather and climate, explicitly solves large spatial distribution phenomena, and parameterizes processes that occur at smaller scales than the model’s spatial resolution. It is run operationally by the Weather Prediction and Climate Studies Center of the National Institute for Space Research – CPTEC/INPE.^19,20,21^

In these studies, the concentrations of the pollutant analyzed were fine particulates, PM_2.5_, which corresponds to around 60% of PM_10_, and exposure was associated with hospitalizations. Temperature is often a control variable in Poisson regression models that analyze the effects of environmental pollution on human health. However, there is a strong connection between meteorological variables and air pollutants.^22^ There is evidence that temperature can modify the effects of exposure to air pollutants such as PM_10_, but this modifying effect appears to be inconsistent.

Shen et al.^22^, using a generalized additive model, reported that exposure to PM_10_ at low temperatures resulted in more pronounced effects on the respiratory system; in their study, men and children aged 15 and under were more vulnerable to low temperatures. Our findings do not coincide with the study above carried out in the Chinese province of Lanzhou, possibly due to the difference between the local average temperature of 11.4°C, ranging between -12.4°C and 29.8°C, much lower than those found in Vale do Paraíba, with an average of 16°C and variation between 2°C and 23°C. Furthermore, PM_10_ concentrations in the Chinese study were around 135 μg/m^3^, five times higher than the values found in Vale do Paraíba.

The ecological type of study may have limitations such as the concentrations of pollutants and the values of meteorological variables being homogeneous in the three municipalities that have no physical barriers between them; another possible limitation lies in the fact that the secondary data analyzed, even from an official source, DATASUS, may contain errors in filling out the diagnoses or the children’s place of residence. Furthermore, it is not possible to identify values of indoor concentrations of pollutants. Additionally, one very important limitation is the ecological fallacy, which is the statistical interpretation of data in which inferences about an individual’s nature are deduced from a population group to which the individual belongs. Also, the hospitalizations, the most serious situation of illness, refer to those carried out by the SUS, not counting those incurred under medical insurance or health plans. It would be interesting if information on the onset of symptoms and outpatient care were available. On the other hand, the approach using conurbated cities as a study site has already been presented as an alternative for evaluating the effects of air pollution on health.^23^

Even with the possible limitations mentioned above, studies with agglomerated cities can reinforce the harmful role of exposure to air pollutants on children’s health, in the case described here, particulate matter. It was possible to estimate the excess number of hospitalizations if these concentrations decreased and also the financial cost for the health system, but not the cost for the hospitalized child and their family.

## Data Availability

The database that originated the article is available with the corresponding author.
